# How pharmacokinetic and pharmacodynamic principles pave the way for optimal basal insulin therapy in type 2 diabetes

**DOI:** 10.1111/j.1742-1241.2010.02470.x

**Published:** 2010-09

**Authors:** S Arnolds, B Kuglin, C Kapitza, T Heise

**Affiliations:** PROFIL Institut für Stoffwechselforschung GmbHNeuss, Germany

## Abstract

This pedagogical review illustrates the differences between pharmacokinetic (PK) and pharmacodynamic (PD) measures, using insulin therapy as the primary example. The main conclusion is that PD parameters are of greater clinical significance for insulin therapy than PK parameters. The glucose-clamp technique, the optimal method for determining insulin PD, is explained so that the reader can understand the important studies in the literature. Key glucose-clamp studies that compare two basal insulin analogues – insulin glargine and insulin detemir – to Neutral Protamine Hagedorn insulin and to each other are then presented. The review further explains how PD parameters have been translated into useful clinical concepts and simple titration algorithms for everyday clinical practice. Finally, the necessity of overcoming patient and/or physician barriers to insulin therapy and providing continuing education and training is emphasised.

**Review Criteria**

Information was gathered from the literature published on PubMed (until 31 October 2009) on the topics of type 2 diabetes and insulin therapy, including basal insulin analogues, glucose clamps, pharmacokinetics and pharmacodynamics.

**Message for the Clinic**

For insulin therapy, pharmacodynamic (PD) assessments have more clinical relevance than pharmacokinetic assessments. The glucose-clamp technique is the gold standard method to determine insulin PD and provides valuable information with respect to onset, peak and duration of action. Glucose-clamp studies and clinical trials demonstrate that once-daily administration of insulin glargine and insulin detemir provides effective blood glucose control and reduces the risk of hypoglycaemic excursions relative to Neutral Protamine Hagedorn insulin. Apart from a theoretical understanding of insulin PD, appropriate education and training of both patient and healthcare worker are essential prerequisites to successful everyday insulin supplementation.

## Introduction

Endogenous insulin secretion, which is tightly regulated in healthy people to maintain euglycaemic plasma glucose levels between 4 and 6 mmol/l, consists of a rather constant, although still pulsatile, basal insulin secretion pattern complemented by markedly increased prandial insulin secretion. The latter depends on individual need and is highly variable in terms of quantity and duration (1). The main role of basal insulin is to limit lipolysis and hepatic glucose production in the fasting state, especially during the night, while ensuring sufficient glucose for cerebral function. The primary task of prandial insulin is to suppress hepatic glucose production and stimulate utilisation of glucose by muscle, thus preventing hyperglycaemia after meals (2).

Numerous efforts have been undertaken by pharmaceutical companies to develop insulin formulations that closely mimic the kinetics of this complex endogenous insulin secretion pattern. Consequently, a variety of insulin analogues are now available for clinical use, including rapid-acting insulin analogues that can be administered before or during meals for prandial control (regular human insulin, insulin lispro, insulin aspart and insulin glulisine), long-acting insulin analogues that can be administered once- or twice-daily for basal insulin supply (NPH insulin, insulin glargine and insulin detemir) and insulin premixes that contain both basal and prandial insulins in a single injection (3). None of the analogue premixes contains NPH.

This pedagogical review describes several key principles of pharmacokinetics (PK) and pharmacodynamics (PD) used in diabetes research. It further describes how these principles can be translated into clinical diabetology. The content has a particular focus on basal insulins for type 2 diabetes, but the concepts are generally applicable for many therapeutics. Our goal has been to address common questions on insulin pharmacology that arise frequently in discussions with general practitioners.

## Question 1: What is the pharmacology of endogenous insulin?

Insulin is secreted from pancreatic β-cells located in the islets of Langerhans and enters the circulation primarily in response to a rise in blood glucose. Apart from stimulating peripheral glucose utilisation in the main insulin-dependent tissues, i.e., skeletal muscles and adipose tissue, insulin antagonises the effect of glucagon in the liver by inhibiting glucose and ketone body production. In addition, because of the anatomy of the blood circulation within the Langerhans islets, insulin directly inhibits glucagon secretion from neighbouring pancreatic α-cells independently of blood glucose. In healthy non-obese adults, insulin is secreted at a basal rate of 0.5–1 U/h (4), resulting in plasma concentrations of 5–15 μU/ml in fasting conditions (5). Within 30–60 min of a meal, insulin levels rapidly increase to peak concentrations of 60–80 μU/ml and return to baseline 2–4 h later (5). In general, obese subjects show much higher basal and postprandial levels.

Once released from β-cells or a subcutaneous depot, insulin clearance appears to be a rather complex process. The liver and kidney are the main sites of insulin degradation, but insulin clearance is probably also mediated by insulin receptor dynamics and depends highly on insulin concentration (6).

## Question 2: What are the limitations of PK parameters relative to PD parameters for understanding therapeutic insulins?

**Figure 1:**
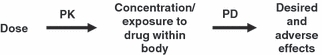
Relationship between pharmacokinetics (PK) and pharmacodynamics (PD)

In general, PK may be regarded as what the body does to a drug, and PD as what a drug does to the body. PK comprises the relationship between drug input – which includes adjustable factors such as dose, dosage form, frequency and route of administration – and the concentration achieved with time. PD, in contrast, comprises the relationship between drug concentration and both the intended and adverse effects produced with time (7). A simplified PK/PD scheme is shown in Figure 1.

**Figure 2:**
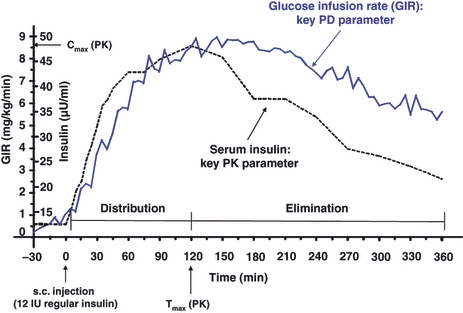
Comparison of pharmacokinetics (PK) (serum insulin concentrations) and pharmacodynamics (PD) (glucose infusion rate) over time after a single subcutaneous injection of insulin. The difference between the two curves illustrates the temporal separation between PK and PD effects

The PD of a drug, i.e., the duration of its biological effects after administration, are the product of numerous target interactions and/or downstream signalling events that occur in multiple cells and organs in response to the drug. The kinetics of these events occurs over an extended time frame relative to that of drug availability. In the specific case of insulins, the temporal separation between the PK and PD profiles (Figure 2) is the result of a series of insulin-specific phenomena, including the fraction and rate of absorption from subcutaneous tissue, the rate of binding to insulin receptors and subsequent induction of metabolic processes, including elimination. Pharmacological profiles of different insulin preparations can only be understood when these PD effects are taken into account. This is especially true when considering the long-acting basal insulins (8).

One PK term in particular, ‘half-life’, often presents significant misunderstandings when considered in relation to insulins. The elimination half-life (*t*_1/2_) is defined as the time necessary for the plasma concentration, as well as the amount of the drug in the body, to fall by one half after the distribution phase has ended and the elimination phase has begun (Figure 2). Thus, it always takes at least four elimination half-lives from the time of peak plasma concentration for a drug to be nearly completely eliminated from the body (50% + 25% + 12.5% + 6.25% = 93.75%). It is important to know the elimination half-life of a drug when designing or prescribing a drug to evaluate its potential for accumulation.

After secretion from the pancreas, the estimated biological half-life of insulin in the blood stream lies between 3 and 10 min. This value, however, is irrelevant for insulin injections in diabetic patients because, as the circulating hormone is cleared from the blood, fresh insulin is continuously released from the subcutaneous depot produced by the injection. For commercial preparations, insulin levels in blood are driven primarily by the more prolonged absorption rate from the depot, which lasts several hours for all insulin formulations available. As a result, the elimination half-life is a more clinically relevant parameter than the biological half-life.

These considerations also resolve what some clinicians may mistakenly view as a paradox regarding long-acting insulins, i.e., that they have elimination half-lives (often denoted in this context as ‘terminal half-lives’) of only 5–7 h, and yet are recommended to be dosed once-daily. The key consideration here is that the elimination half-life only applies after the drug has been fully distributed. As a result of the slow absorption of these agents, the distribution phase can last for many hours. In the case of insulin detemir, for instance, the distribution phase in adults lasts approximately 8 h (9). The remaining 16 h in the 24-h period is covered by 2–3 elimination half-lives. Given the temporal delay for PD effects (Figure 2), insulin levels remain high enough across a 24-h period to support once-daily administration.

Finally, another technical issue concerning insulin PK is the lack of methodologies for comparing absolute values of serum concentrations of different insulins, including basal insulin preparations. Well-established assays are available for NPH insulin and insulin detemir (10), but for insulin glargine, neither commercial assays nor optimal alternative methods are available (11). Consequently, PK parameters published for insulin glargine are based on indirect measures and may, therefore, be less precise.

## Question 3: What is the best method to investigate insulin PD?

The following challenges have to be borne in mind when assessing PD measurements of insulins, or other blood glucose-lowering agents, in human subjects. First, the initial/basal blood glucose level prior to the administration of insulin is of importance, as the lower the blood glucose, the higher the insulin sensitivity and *vice versa*. Second, there is a high inter- and intra-individual variability with respect to both the effects of intake of a specific amount of carbohydrates and a given dose of insulin (this necessarily imposes limitations on oral glucose tolerance and standard meal tests, as well). Third, hypoglycaemia and consequent counter-regulation following an insulin injection limit experiments in healthy volunteers.

The solution to the first challenge is to induce and maintain a fixed initial blood glucose level in study subjects so that they have comparable metabolic situations and insulin sensitivities at the beginning of the study. The solution to the second is to ensure that subjects fast during the investigational period to avoid problems related to food intake. Finally, the solution to the third is to infuse glucose into patients who have just received exogenous insulin to prevent hypoglycaemia and counterregulation. This is the approach taken in glucose-clamp experiments.

The glucose-clamp technique is the gold standard method for investigating PD profiles of insulin preparations (12). In clamp studies, insulin is injected into subjects, and the subsequent PD effects are investigated by preventing the expected decrease in blood glucose concentration with a variable glucose infusion that ‘clamps’ the blood glucose to a predetermined level. A plot of the amount of glucose infused over time, expressed as glucose infusion rate (GIR) in mg/kg/min necessary to maintain blood glucose at the clamp level, accurately reflects the PD effect of the study insulin (13). Figure 2 shows the PK (serum insulin concentration) and PD (GIR) profiles of a soluble insulin during a euglycaemic glucose clamp. Note the temporal shift between the PK and PD curves.

**Figure 3:**
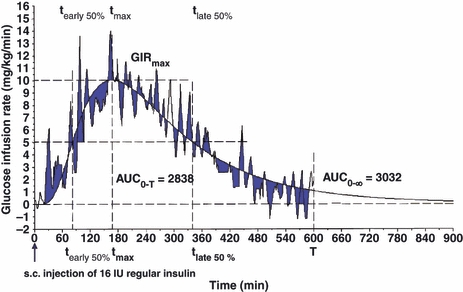
Key pharmacodynamics (PD) parameters from a glucose-clamp experiment

**Figure 4:**
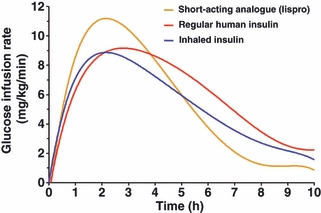
Glucose infusion rates in glucose-clamp experiments on rapid-acting insulins (64)

**Figure 5:**
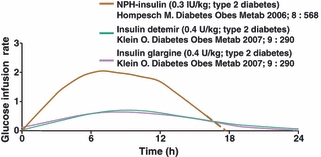
Glucose infusion rates in glucose-clamp experiments on long-acting insulins

A number of parameters of interest can be derived from PD profiles (Figure 3). These include the area under the curve (AUC), i.e., the overall glucose-lowering effect/glucose disposal, and GIR_max_ and *t*_max_, i.e., the magnitude and time of peak effect, respectively. Also relevant to patients are the time to 50% of maximum effect (early *t*_50%_), i.e., the onset of action of the respective insulin, and the time when the maximum effect has fallen again by 50% (late *t*_50%_), i.e., the vanishing effect/end of action. For basal insulins, important parameters include: duration of action, i.e., the time period between insulin injection and end of action; and the end of action, i.e., the time from injection of the study insulin to an increase in serum glucose concentration above a predetermined value (often 8.3 mmol/l) (13). The duration of action can only be measured reliably in people with type 1 diabetes. In such patients, a declining metabolic effect of the study insulin causes an immediate rise in blood glucose. By contrast, in healthy people or patients with type 2 diabetes, duration of action can be overestimated because of endogenous insulin secretion. Duration of action is of relevance in patients who are deciding whether to inject basal insulin once- or twice-daily. Figures 4 and 5 provide GIR curves on rapid-acting and long-acting insulins.

Glucose clamps can be performed ‘manually’ or, more accurately, by an automated procedure using a Biostator (MTB Medizintechnik, Ulm, Germany), which measures the arterialised blood glucose concentration every minute and adjusts the GIR according to a negative feedback algorithm based on the deviations of the glucose measurements from the clamp glucose target level (8).

## Question 4: What should the ‘ideal’ basal insulin look like and how do current products compare?

From a pharmacological point of view, key characteristics of an ‘ideal’ basal insulin should include:

- The PD profile should be flat (peakless) and should be associated with a low risk of hypoglycaemia (which might be caused by nocturnal peak activity several hours after injection).
- The duration of action should be around 24 h to control fasting plasma glucose (FPG) with just one injection per day.
- Variability within individual patients should be low, meaning that identical doses of insulin administered to the same patient on different occasions should lead to identical and predictable effects, thus lowering the risk of hypoglycaemia or hyperglycaemia.

The next sections describe current basal insulin preparations in terms of these ideal characteristics.

### NPH insulin

Neutral Protamine Hagedorn (NPH), introduced by Hagedorn in 1946, contains protamine and insulin in ‘isophane’ amount, i.e., there is neither an excess of protamine nor of insulin. The addition of zinc at low concentrations allows the protamine to form crystals with insulin at neutral pH. NPH insulin is a suspension and must be properly re-suspended before injection, which may contribute to increased variability when not performed carefully (14). A representative glucose-clamp study (15) described the PK and PD of NPH insulin and highlighted its limitations: a short duration of action (14 ± 3 h), a significant peak at 4.5 ± 0.5 h and a high inter-individual variability.

### Insulin glargine

In 2000, insulin glargine became the first basal insulin analogue available. Insulin glargine differs from human insulin at position A21 of the A chain (substitution of asparagine with glycine) and position B31 and B32 of the B chain (addition of two arginines). These changes shift the isoelectric point from pH 5.4–6.7 (16). Insulin glargine is injected as a clear acidic solution (pH 4), which forms microprecipitates that must dissolve before absorption can take place. Precipitation and slow re-dissolution are inherently associated with substantial variability. Nonetheless, the time–action profile of insulin glargine is flatter and of longer duration compared with NPH insulin (15).

### Insulin detemir

Insulin detemir differs from human insulin; in that, threonine at position B30 has been removed and that lysine at B29 has been acylated with a 14-carbon fatty acid (myristic acid). The prolonged duration of action for insulin detemir is attributable to a combination of increased self-association (hexamer stabilisation and hexamer–hexamer interaction) and albumin binding because of the acylation. Insulin detemir is highly albumin-bound (98.8%) in the interstitial fluid and in plasma (17). The analogue is supplied as a clear neutral solution and remains in solution in the subcutaneous depot, in the circulation and in the target tissues until interaction with the insulin receptor. Absorption of insulin detemir is therefore dependent on neither appropriate re-suspension before injection and dissolution of crystals, as is the case with NPH insulin, nor on formation and re-dissolution of microprecipitates, as is the case for insulin glargine. Insulin detemir has a much flatter and longer time–action profile compared with NPH insulin (13), as well as reduced variability compared with insulin glargine (13).

## Question 5: What are the results from PD glucose-clamp studies with basal insulin analogues?

The information of most clinical relevance for basal insulins involves the critical issues of flatness, duration of action and variability. For flatness and duration of action, one needs to look at the GIR curves from a number of published glucose-clamp studies (15,18–22), which have been summarised in a recent review (13). For insulin glargine, all available studies, with one exception (15), showed a very gentle rise and fall over time, indicating a relatively flat activity profile with some evidence of a very broad, albeit small, peak. Mean duration of action was close to 24 h in patients with type 1 diabetes and at least 24 h in people with type 2 diabetes around the clinically relevant dose range of 0.4 U/kg. The same conclusions – a much flatter profile than NPH insulin and duration of action of around 24 h – were consistently proven for insulin detemir by all studies, apart from one outlier (22).

A recent glucose-clamp study (23) compared duration of action of insulin glargine and insulin detemir after single and repetitive doses (administration over 7 days) in subjects with type 1 diabetes. Both basal insulins had durations of action approximating 24 h, although the duration of action after a single dose was shorter for insulin glargine than insulin detemir (19.8 ± 14.4 h vs. 25.9 ± 4.6 h, respectively), whereas the durations of action after repetitive doses were 23.3 ± 4.9 h for insulin detemir and 27.1 ± 7.7 h for insulin glargine. In addition, two other clamp studies in type 1 (21) and type 2 diabetes (20) showed that the duration of action of basal insulin analogues was dose-dependent, i.e., higher doses resulted in longer durations of action, as is observed for all insulins and, indeed, for all pharmaceuticals.

‘Within-subject variability’ is defined as the degree of difference in the glucose-lowering effect from one injection to another within the same patient. For basal insulin analogues, this relates to the consistency of the 24-h PD profile from one injection to the next, which can only be assessed in repeat clamp studies. Such a study (19) was performed in subjects with type 1 diabetes who underwent 24-h glucose-clamp analyses with insulin detemir (*n* = 18), insulin glargine (*n* = 16) or NPH insulin (*n* = 17). Each subject received four single subcutaneous doses of each basal insulin on four different clamp days; all insulins were administered at the same dose (0.4 U/kg). Insulin detemir was associated with significantly less within-subject variability than both NPH insulin and insulin glargine. The coefficients of variation for the PD end-point GIR–AUC_(0–24 h)_ were 27% for insulin detemir, 48% for insulin glargine and 68% for NPH insulin. Lower within-subject variability for insulin detemir was also confirmed in patients with type 2 diabetes in another clamp study (20), although the latter study had the limitation that no replicate experiments with identical doses were conducted, so the results had to be dose-corrected to investigate variability.

## Question 6: How do phase 1 and 2 glucose-clamp studies predict clinical outcome data?

Ideally, results from glucose-clamp studies on basal insulin analogues should be applicable in the clinic when one wants to determine the optimal balance between metabolic control and hypoglycaemia. A basal insulin analogue, when compared with NPH insulin, should result in: comparable metabolic control with less hypoglycaemic episodes; improved metabolic control with comparable hypoglycaemic events; or, in the best case scenario, improvement in both. Does this actually hold true in phase 3 trials?

### Insulin glargine vs. NPH insulin

In a number of clinical trials, patients with type 2 diabetes exhibited comparable HbA_1c_ reductions (24–27) and rates of achieving target HbA_1c_ goals (≤ 7.0%) (24,27) when administered insulin glargine or NPH insulin. Moreover, there was a consistent and significant reduction in hypoglycaemia risk with insulin glargine compared with NPH insulin for both overall symptomatic (11%, p = 0.0006) and nocturnal (26%, p < 0.0001) hypoglycaemic events (24,28). In one meta analysis (24), risks of overall severe and severe nocturnal hypoglycaemia were reduced by 46% (p = 0.0442) and 59% (p = 0.0231), respectively, although another (25) did not find significant differences in confirmed or severe episodes. One study (27) showed a lower rate of hypoglycaemic excursions and less variability of FPG in subjects taking insulin glargine compared with NPH insulin.

### Insulin detemir vs. NPH insulin

Large randomised clinical trials comparing insulin detemir and NPH insulin have demonstrated that glycaemic control with insulin detemir was similar to, or better than, NPH insulin (29,30). Use of insulin detemir was associated with a significant reduction in the risk of nocturnal hypoglycaemia in the majority of studies (31–37) [up to 87% or 90% risk reduction compared with NPH insulin (35,38)].

Insulin detemir therapy also provided more predictable glycaemic control and less intra-patient variability than NPH insulin in both type 1 and type 2 diabetes (29,32,36,38–42). In most trials (32,36,38–42), intra-patient variation in self-measured FPG was significantly lower with insulin detemir than NPH insulin. In addition, nocturnal plasma glucose profiles were more stable, with lower glucose fluctuations (32,36). Increased intra-patient variability with insulin therapy may increase the risk of hypoglycaemia, as was shown from a meta analysis (43) and two further publications (44,45). These studies showed a positive correlation between the incidence of hypoglycaemia and the coefficients of variation in FPG: a reduction of 2.7% in the within-patient variation in FPG resulted in 2.77% fewer hypoglycaemic events per subject per year, independent of the type of treatment (43). Thus, a decrease in intra-patient variability, as seen with insulin detemir, is worth noting – not only from glucose-clamp study results, but also from the clinical data described above.

### Insulin glargine vs. insulin detemir

Four head-to-head clinical trials comparing insulin glargine and insulin detemir have been published, including one study in type 1 diabetes (46) and three in type 2 diabetes (47–49). One 52-week treat-to-target trial evaluated both analogues in a basal-bolus regimen with mealtime insulin aspart in 319 type 2 subjects treated with oral antidiabetic drugs (OADs) or insulin, with or without OADs (47). A second 52-week study compared insulin detemir with insulin glargine administered as add-on therapy to OADs in 582 insulin-naïve subjects with type 2 diabetes (48). In both trials, insulin detemir and insulin glargine were equally effective in optimising HbA_1c_. The two analogues were associated with comparable hypoglycaemia risks and variabilities, but insulin detemir therapy was associated with less weight gain. Another head-to-head study (49) was a double-blind, randomised, crossover study in subjects with type 2 diabetes that included continuous glucose monitoring after careful insulin titration over several days. In this study, once-daily dosing of insulin detemir provided glycaemic control similar to that of insulin glargine over a 24-h period.

## Question 7: Treatment strategies with basal insulin analogues in type 2 diabetes: what is the evidence from clinical trials?

According to a consensus statement from the American Diabetes Association and the European Association for the Study of Diabetes (50), initiation of insulin therapy in patients with type 2 diabetes should start with either bedtime intermediate-acting or bedtime or morning long-acting insulin (10 units or 0.2 U/kg). A recent review on insulin therapy in type 2 diabetes (51) also concluded that once-daily basal insulin added to oral medication is an ideal starting point. However, all next steps, from one to two or even more daily injections, are controversial and should be considered carefully with the respective patient. An important issue is the early intensification of insulin therapy to achieve and keep target HbA_1c_ values. In the 3-year 4-T study (52–54) investigating complex insulin regimens in type 2 diabetes, 68–82% of patients received an additional type of insulin to achieve a median HbA_1c_ level of 6.9% and, thus, needed ‘complex’ regimens.

A recent meta analysis on optimal insulin regimens in type 2 diabetes (55) found greater HbA_1c_ reductions in insulin-naïve patients treated with biphasic or prandial insulin, compared with basal insulin [0.45% (p = 0.0006) and 0.45% (p = 0.02), respectively], but with lesser reductions of fasting glucose [0.93 mmol/l (16.8 mg/dl; p = 0.01) and 2.20 mmol/l (39.7 mg/dl; p < 0.00001), respectively]. Moreover, minor hypoglycaemic events were inconsistently reported as either higher than or equivalent to basal insulin, and there was greater weight gain with prandial compared with basal insulin (1.86 kg, p = 0.0006).

In the 3-year 4-T study (52,53), 708 subjects with type 2 diabetes and inadequate glycaemic control on metformin and sulfonylurea were randomly assigned to receive prandial insulin aspart, basal insulin detemir or biphasic insulin aspart. Starting in the second year, sulfonylureas were replaced by an additional insulin (basal insulin added to prandial insulin, prandial insulin three times daily added to basal insulin and prandial insulin at lunch added to biphasic insulin) if HbA_1c_ levels were above 6.5%. An important feature of this study was its long duration and the standardisation of insulin regimens.

Less than 45% of all patients reached the HbA_1c_ target of ≤ 6.5% (and even less than one-third in the biphasic group). In addition, there were striking differences in outcomes between the first and third years. Although the basal regimen was least successful in the first year, it was effective after 3 years, probably because of a progressive increase of the insulin dose. The basal insulin regimen, which was equivalent to the other regimens after the first year in patients with HbA_1c_ values of 8.5% or less, was superior to both prandial and biphasic insulin after 3 years in terms of weight gain and the rate of hypoglycaemia. Thus, the 4-T study supports the initiation of treatment with basal insulin.

This conclusion is consistent with the concept that fasting hyperglycaemia contributes more than postprandial hyperglycemias to HbA_1c_ levels. As shown by Monnier, the relative contribution of fasting hyperglycaemia to HbA_1c_ levels increased gradually as diabetes worsened, whereas that of postprandial glucose excursions were predominant in relatively well-controlled patients (56). Thus, it makes sense to focus on FPG during insulin initiation. Several studies investigated treatment algorithms for insulin glargine (27,57–59) and insulin detemir (60,61) (Table 1). A number of these algorithms depend on patient-managed self-titration, which has proven to be safe and efficacious.

**Table 1:** Treatment algorithms for insulin glargine and insulin detemir in type 2 diabetes

Insulin glargine (GLAR)			
Author	Study/number of patients	Intervention	Algorithm
Riddle(*Diabetes Care* 2003)	24-Week treat-to-target trial*n* = 756	GLAR or NPH once daily at bedtime	Starting dose: 10 U/dayIf mean FPG (mmol/l) over previous 3 days:≥ 5.6 to < 6.7 → 0–2 U ↑≥ 6.7 to < 7.8 → 2 U ↑≥ 7.8 to < 10.0 → 4 U ↑≥ 10.0 → 6–8 Uand no PG < 4.0 mmol/l
Davies(*Diabetes Care* 2005)	ATLANTUS*n* = 4961	GLAR;clinic- vs. patient-managed dose titration	Clinic-managed titration: as in Riddle studyPatient-managed titration:2 IU↑ every 3 days, no PG < 4 mmol/l
Yki-Järvinen(*Diabetologia* 2006)	36-Week LANMET trial*n* = 110	Bedtime GLAR vs. NPH	Patient-managed titration:2 IU↑ every 3 days, if FPG above 4.0–5.6 mmol/l, stop titration if ≥ 1 hypoglycaemic event

Insulin detemir (DET)			

Author	Study details	Intervention	Algorithm details

Meneghini(*Diabetes Obes Metab* 2007)	PREDICTIVE 303 Trial*n* = 5604	DET as add-on to OAD or as replacement of prestudy insulin	Patient-managed titration:every 3 days: mean adjusted FPG (mmol/l)< 4.4 → 3 U↓4.4–6.1 → no change> 6.1 → 3 U↑vs. physician-managed titration:according to standard of care
Blonde(*Diabetes Obes Metab* 2009)	20-Week TITRATE Trial*n* = 244	DET once daily, insulin-naïve patients on OAD	Two FPG (mmol/l) titration targets:(1) 3.9–5.0(2) 4.4–6.1Titration as in PREDICTIVE

NPH, Neutral Protamine Hagedorn; FPG, fasting plasma glucose; OAD, oral antidiabetic drugs.

## Question 8: Which factors – keeping insulin PD on one’s mind – have an impact on appropriate (basal) insulin substitution in ‘real life’?

Patient and/or physician barriers to insulin initiation or intensification often need to be overcome before insulin therapy can be implemented successfully (62,63). One of the key patient barriers is fear of hypoglycaemia, which can be managed by appropriate training, introduction of blood glucose self-monitoring (50,62), use of basal insulin analogues (62) and translation of PD concepts into practical clinical dosing regimens. Decisions on insulin dose adjustments should be knowledge based. Thus, the patient should be enabled to differentiate whether a high fasting glucose value resulted from deficiency in insulin, diet violation the prior evening or from nocturnal hypoglycaemia and counter-regulation. Training should be provided to deal with the specific situations of illness, fever and demobilisation and unplanned exercise or extra meals. General practitioners who face time constraints or lack familiarity with tailored insulin treatment should obtain continuing education and should have a hot line to specialists for rapid consultation (63).

## Conclusions

Measurements of serum insulin concentrations in patients with diabetes do not add value to clinical practice. Moreover, the biological half-life of insulin is unimportant to either its efficacy or duration of action. Instead, the PD profile is far more informative than the PK profile in terms of determining dosing frequency and expectations of efficacy over a given period of time.

The optimal method for assessing PD parameters is the glucose-clamp technique. Results of studies using this technique have demonstrated that insulin glargine and insulin detemir have flatter, but not completely peakless, time–action profiles compared with NPH insulin. The basal insulin analogues also have comparable durations of action of around 24 h, with less intra-subject variability for insulin detemir. These conclusions have been confirmed in clinical trials, which have shown that once-daily dosing of insulin glargine and insulin detemir is possible and that these two basal analogues provide equal metabolic control relative to NPH insulin in terms of HbA_1c_. Compared with NPH insulin, use of insulin glargine and insulin detemir is associated with less hypoglycaemic events (particularly nocturnal ones), lower FPG and – with a slight advantage for insulin detemir – less intra-subject variability of fasting glucose. Patients can self-titrate the two basal insulin analogues effectively and safely by means of simple titration algorithms.

Finally, in addition to a sufficient knowledge about insulin PD, appropriate education, empowerment and training of both the patient and the healthcare worker are essential to overcome potential barriers to insulin therapy, deal with specific situations and successfully implement everyday insulin supplementation. These steps, if followed through appropriately, should facilitate patient care and improve quality of life for patients with type 2 diabetes.
